# Sexually transmitted infections among at-risk women in Ecuador: implications for global prevalence and testing practices for STIs detected only at the anorectum in female sex workers

**DOI:** 10.1136/sextrans-2023-056075

**Published:** 2024-08-07

**Authors:** Luz Marina Llangarí-Arizo, Claire Elizabeth Broad, Liqing Zhou, Miguel Martin Mateo, Cristina Izquierdo Moreno, Marcelo Moreno Cevallos, Philip J Cooper, Natalia Romero-Sandoval, Syed Tariq Sadiq

**Affiliations:** 1 Escuela de Medicina, Universidad Internacional del Ecuador, Quito, Ecuador; 2 Institute for Infection and Immunity, St George's University of London, London, UK; 3 Autonomous University of Barcelona, Barcelona, Spain; 4 Red Internacional Grups de Recerca d'América i África Llatines-GRAAL, Barcelona, Spain

**Keywords:** PUBLIC HEALTH, Epidemiology, PCR

## Abstract

**Abstract:**

**Objectives:**

Anorectal sexually transmitted infections (STIs) such as *Chlamydia trachomatis* (CT) and *Neisseria gonorrhoeae* (NG), present treatment challenges, potentially increase antibiotic resistance selection and if undetected may facilitate onward transmission. However, there are limited global prevalence data for anorectal STIs. We conducted a cross-sectional study to assess the prevalence and risk factors of non-viral genital and extragenital STIs in female sex workers (FSW) and female non-sex workers (NSW) in Ecuador.

**Methods:**

250 adult street and brothel FSWs and 250 NSWs, recruited from settlements in north-west Ecuador provided oropharyngeal and vulvo-vaginal swabs (VVS) as well as socio-demographic data. FSWs also provided anorectal swabs. PCR was used to detect CT, NG, *Mycoplasma genitalium* (MG) from all swabs and additionally *Trichomonas vaginalis* (TV) from VVS. Risk factors were analysed using logistic regression.

**Results:**

Prevalence of FSW vaginal, anorectal and oropharyngeal infection was 32.0% (95% CI 26.5% to 38.0%), 19.7% (95% CI 15.1% to 25.2%) and 3.2% (95% CI 1.6% to 6.2%), respectively, with most vaginal infections being TV (23.4%; 95% CI 18.5% to 29.2%). Overall FSW STI prevalence, at any anatomical site was 39.7% (95% CI 33.8% to 46.1%), with 12.1% (95% CI 8.5% to 16.9%) of infections detected only at the anorectum. Of all the CT and/or NG infections, 64.4% (95% CI 50.4% to 78.4%) were detected only at the anorectum. STI prevalence in NSWs in the vagina and oropharynx were 5.6% (95% CI 3.4% to 9.2%) and 0.8% (95% CI 0.2% to 2.9%), respectively, with most vaginal infections being MG (3.2%; 95% CI 1.6% to 6.2%). In multivariable analysis, risk factors among brothel-based FSWs for having an anorectal STI were vaginal CT, NG or MG (p<0.001), vaginal TV (p=0.029) and being ‘in a relationship’ (p=0.038).

**Conclusions:**

High prevalence of CT and NG detected only at the anorectum in these FSWs indicate the possibility of missing significant infections if providing only genital testing and calls for greater research into the potential impact on global STI estimates if extragenital infections among at-risk women are not identified.

**WHAT IS ALREADY KNOWN ON THIS TOPIC**

- Prevalence studies of sexually transmitted infections (STIs) across Ecuador and particularly among female sex workers (FSWs) focus on HIV or syphilis infection. Evidence for extragenital STIs (ie, infection of the pharynx and anorectum) among sex workers and non-sex workers is limited despite the disproportionate impact of these infections among at-risk communities.

**WHAT THIS STUDY ADDS**

- We present prevalence and risk factors associated with vaginal and anorectal non-viral STIs among FSWs recruited from three characteristically different regions of Ecuador, and vaginal STI prevalence data from non-sex workers from the same regions. This is the first study in Ecuador that establishes genital and extragenital non-viral STI prevalence of FSWs and highlights the high proportion of exclusive anorectal infection.

**HOW THIS STUDY MIGHT AFFECT RESEARCH, PRACTICE OR POLICY**

- These findings contribute to our understanding of infection prevalence, particularly of extragenital infections, among FSWs. We identify the potential need for extragenital testing among FSWs in Ecuador and if globally applicable, highlight the importance of multi-anatomical site testing, for female at-risk populations.

## Introduction

 Sexually transmitted infections (STIs) with *Chlamydia trachomatis* (CT), *Neisseria gonorrhoeae* (NG), *Trichomonas vaginalis* (TV) and *Mycoplasma genitalium* (MG), can result in serious reproductive health sequelae, including pelvic inflammatory disease and infertility.^1 2^ Global prevalence of female urogenital STIs is estimated to be 5.3%,^3^ 3.1%^1^ and 0.9%^2^ for TV, CT and NG, respectively. Among at-risk populations such as female sex workers (FSWs),^4^ urogenital STI estimates vary, with pooled prevalence for TV at 16%^5^ and CT, NG and MG prevalence between 10%–16%,^6^ 1%–29%^7^ and 13%–26%,^8^ respectively.

However, few prevalence data are available for female oropharyngeal and anorectal STIs, which are often asymptomatic and may alter clinical management.^7 9^ Testing for female extragenital STIs, occurs usually, in the context of a history of anal or oral sex.^10^ In high-income countries, female anorectal CT infection may be high in those with concurrent urogenital infection.^11^ Globally, anorectal NG infection is reported at lower prevalence (0–17%) compared with CT (7–17%)^12 13^ and possibly more associated with reported anal intercourse.^13^ For anorectal MG, data in women are scarce, with one study describing 22% prevalence among ‘high-risk’ individuals.^14^ Interestingly, despite being the most common non-viral STI among women globally, anorectal TV prevalence studies have only been reported among men-who-have-sex-with-men.^15^

Across Latin America, few STI prevalence data are available, not only for FSWs but also non-sex workers (NSWs).^16^ In Ecuador, FSWs work in brothels or solicit clients on the streets, moving locations regularly, have high client numbers (personal communication with Romero, N) and risky sexual behaviours such as unprotected anal intercourse.^16^ We estimated urogenital and extragenital prevalence and associated risk factors for these STIs among FSWs and NSWs in north-west Ecuador.

## Methods

### Study design

#### Engagement

To establish appropriate ways to conduct this study in different settings such as brothels, we held workshops in the three recruitment locations with key stakeholders, including the Ministry of Health, local clinicians, brothel and hotel owners and representatives of the FSW worker associations. General interest in the study was positive, particularly how knowledge of STI prevalence in these key populations might improve general and reproductive health.

#### Design

A cross-sectional study was conducted among FSWs and NSWs in three localities in north-west Ecuador between November 2018 and April 2019: Location A in Imbabura province, a small rural town (~7000 inhabitants) and a larger town, Location B (~30 000 inhabitants) in Esmeraldas province. Both towns had high levels of poverty, defined by household income insufficient to meet basic needs and access only to basic healthcare centres.^17^ An additional location was included for FSW recruitment, Location C (~450 000 inhabitants), a densely populated commercial city in Santo Domingo province, with access to medium-level healthcare.^17 18^

We aimed for a sample size of 250 FSWs, assuming FSW urogenital STI rates,^16^ of 18% for any of CT, NG, MG and TV infections, giving 95% precision estimates of 13.7%–23.2% for these STIs. We aimed also to recruit a convenience sample of 250 NSWs by comparison.

### Participant data and clinical samples collection

FSWs were approached by two trained female researchers either in brothels or via FSW associations. NSWs were invited onto the study while waiting in primary healthcare units by two trained female researchers. Advertisement of the study in educational institutions and commercial areas was also used to engage NSWs but recruitment was undertaken confidentially within health centres. Eligibility criteria for all participants included: aged ≥18 years, not pregnant nor menstruating and willing to provide at least one sample.

After obtaining informed written consent, the following samples were collected by a trained researcher within private brothel rooms, hotels in which they worked (street-based FSWs) and health centres for NSWs: vulvovaginal swabs (VVS), oropharyngeal and anorectal swabs (the latter for FSWs only) and blood. Both VVS and anorectal swabs, taken without the use of a speculum or proctoscope, were stored in phosphate-buffered saline and frozen at −20°C before analysis (see online supplemental material 1 for swab protocols). Following discussions with local representatives, there was an agreement from the study team that NSWs would be uncomfortable providing anal swabs or answering questions regarding anal sex; thus these were omitted from the study.

For all participants a validated behavioural questionnaire^19 20^ was adapted to include factors, which emerged from our previous work^16^ was administered face-to-face, using KoboToolbox software (V.1.14.Oa and V.1.25.1). Questionnaire variables included socio-demographic, behavioural factors and clinical history. Discussions during workshops highlighted that ‘intravaginal cleaning’ and ‘intravaginal insertion’ practices were frequently undertaken by the local population. Intravaginal cleaning is defined as washing around the vulva and in the vagina with products such as toothpaste or alcohol, while intravaginal insertion includes using products such as clotrimazole and gentamycin cream added to cloth and retained in the vaginal for an unknown amount of time. As these practices were undertaken for ‘hygiene purposes’ and not to treat symptoms or disease, this was not considered ‘self-medication’. Online supplemental table A1 provides definitions for all variables.

### Laboratory testing

DNA was extracted using the PureLink Genomic DNA Mini Kit (Thermo Fisher Scientific, USA). For vaginal and oropharyngeal DNA extracts, PCR was performed using Applied Biosystems 7500 Fast Real-Time PCR System (Thermo Fisher Scientific, USA) to test for CT, NG, MG and TV (vaginal only). Primer and probe sequences and cycling conditions are shown in online supplemental material 1. Anorectal DNA extracts were stored at −20°C, until transferred to UK on dry ice and tested for CT, NG and MG with the same kit and conditions, using the Bio-Rad CFX-96 Real time PCR system (Bio-Rad Laboratories, USA).

Some oropharyngeal and vaginal DNA extracts were re-tested in the UK for quality control purposes and due to discrepant results, all vaginal DNA extracts were re-tested for TV, using Luna Universal Probe qPCR Kit (New England Biolabs, USA). Blood sample results were excluded from these analyses.

### Data analysis

After data validation and cleaning, analyses were performed using IBM SPSS V.24 and R V.4.1.3. Study data were expressed as frequencies and percentages for categorical variables and medians with IQRs for continuous variables. Proportions, and 95% CIs, for CT, NG, MG and TV infections were derived for each anatomical site. Pathogen co-infection was defined as positive for two or more of these STIs in the same sample from one anatomical site. Multisite infection was defined as one or more of these STIs in at least two anatomical sites. Infections detected only at the anorectum were any individuals testing positive for one or more STIs only in the anorectum. For example, a positive anorectal test but negative tests from vaginal and oropharyngeal samples.

Comparisons of categorical and continuous variables were done using χ^2^ and Kruskal-Wallis test, respectively. Because the two groups of FSWs (brothel-based workers (Locations A and B), and street-based workers (Location C)) represented distinct populations, separate analyses of risk factors for vaginal and anorectal STIs were undertaken. Following disaggregation of FSWs, the sample size of street-based FSWs was too small for risk factor analysis and was omitted from this study. Risk factors for genital and extragenital STIs among brothel workers were determined using the generalised linear model function within R (V.4.1.3). Any factors with a p<0.05 were taken forward for independent multivariable analysis.

## Results

### Participant demographics and social characteristics

Of 264 FSWs approached, 250 were recruited (14 declined citing ‘lack of time’, embarrassment or ‘shame’ about anorectal sampling): all 250 provided vaginal, 249 oropharyngeal and 239 anorectal swabs. Of 257 NSWs approached, 251 were recruited (6 declined a vaginal swab) of whom 250 provided vaginal and oropharyngeal swabs.

Of the FSWs recruited, 86.8% were Ecuadorian, 8.4% Venezuelan and 4.8% Colombian (table 1); all NSWs were Ecuadorian. FSWs were of similar age and level of education compared with NSWs (median age 29 (IQR 24–32) vs 30 (IQR 25–35), respectively). Street-based FSWs were older (42 (IQR 33–55), less likely to have travelled for sex work, more likely to have reached secondary level education and had more children. Brothel-based FSWs had greater weekly income for sex work compared with street-based FSWs (US$200 (IQR 100–300) vs US$60 (IQR 40–150), p<0.0001). There were no differences in characteristics of brothel-based FSWs between Locations A and B (table 1). Online supplemental table S1, provide detailed demographic and behavioural characteristics of NSWs.

**Table 1:** Socio-demographic, STI history, clinical history and behavioural variables of female sex workers according to the location of the questionnaire

	Location A	Location B	Location C	Overall	Differences observed between locations
	(n=63)	(n=142)	(n=45)	(n=250)	
Median age (IQR)	26 (22–32)	28.5 (25–35)	42 (33–55)	29 (24–32)	p<0.001
Median years as a sex worker (IQR)	2.0 (1.0–7.0)	4.5 (1.7–10.0)	22.0 (7.0–32.5)	5.0 (2.0–13.0)	p<0.001
Country of origin (%)					
Ecuador	59 (93.7)	117 (82.4)	41 (91.1)	217 (86.8)	p=0.057
Other	4 (6.3)	25 (17.6)	4 (8.9)	33 (13.2)	
Travel within Ecuador for sex work (%)					p<0.001
Yes	61 (96.8)	131 (92.3)	15 (33.3)	207 (82.8)	
No	2 (3.2)	11 (7.7)	30 (66.7)	43 (17.2)	
Level of education (%)*					p<0.001
Uneducated (illiterate)	6 (9.5)	0 (0)	6 (13.3)	12 (4.8)	
Primary	36 (57.1)	80 (56.3)	11 (24.4)	127 (50.8)	
Secondary	19 (30.2)	56 (39.4)	26 (57.8)	101 (40.4)	
Higher	2 (3.2)	6 (4.2)	2 (4.4)	10 (4.0)	
Number of children (%)					p<0.001
0	9 (14.3)	18 (12.7)	3 (6.7)	30 (12.0)	
2 January	31 (49.2)	62 (43.7)	8 (17.8)	101 (40.4)	
5 March	19 (30.2)	57 (40.1)	18 (40.0)	94 (37.6)	
8 June	4 (6.3)	4 (2.8)	13 (28.9)	21 (8.4)	
10 September	0 (0)	1 (0.7)	3 (6.7)	4 (1.6)	
Weekly income from sex work US$ (%)					p<0.01
NA	0 (0)	1 (0.7)	0 (0)	1 (0.4)	
0–100	7 (11.1)	16 (11.3)	25 (55.6)	48 (19.2)	
100–250	34 (54.0)	64 (38.7)	12 (26.7)	110 (44.0)	
250–500	14 (22.2)	48 (40.1)	6 (13.3)	68 (27.2)	
500+	8 (12.7)	13 (9.2)	2 (4.4)	23 (9.2)	
Median clients per week (IQR)	25 (15–37.5)	36 (20–50)	10 (6–30)	30 (15–40)	p<0.0001
**Clinical and behavioural characteristics**					
Any vaginal symptoms† (%)					p=0.114
Yes	53 (84.1)	124 (87.3)	35 (77.8)	212 (84.8)	
No	10 (15.9)	18 (12.7)	10 (22.2)	38 (15.2)	
Previous STI (in lifetime) (%)					p<0.001
Yes	4 (6.3)	14 (9.9)	20 (44.4)	38 (15.2)	
No	59 (93.7)	128 (90.1)	25 (55.6)	212 (84.8)	
Previous treatment for suspected STI (in lifetime) (%)					p<0.001
Yes	3 (4.8)	14 (9.9)	20 (44.4)	37 (14.8)	
No	60 (95.2)	128 (90.1)	25 (55.6)	213 (85.2)	
Intravaginal washing or douching (%)					p=0.002
Yes	55 (87.3)	118 (83.1)	28 (62.2)	201 (80.4)	
No	8 (12.7)	24 (16.9)	17 (37.8)	49 (19.6)	
Intravaginal insertion (%)					p=0.636
Yes	23 (36.5)	21 (14.8)	2 (4.4)	46 (18.4)	
No	11 (17.5)	46 (32.4)	19 (42.2)	76 (30.4)	
Unknown	29 (46.0)	75 (52.8)	24 (53.4)	128 (51.2)	
HIV status at last test					p=0.591
Positive	1 (1.6)	1 (0.7)	0 (0)	2 (0.8)	
Negative	60 (95.2)	137 (96.5)	45 (100.0)	242 (96.8)	
Did not want to know.	1 (1.6)	0 (0)	0 (0)	1 (0.4)	
No results yet	0 (0)	1 (0.7)	0 (0)	1 (0.4)	
No answer	1 (1.6)	3 (2.1)	0 (0)	4 (1.6)	

* Level of education refers to the percentage of individuals who attended school at each level, but may not have completed that stage.

† Vaginal symptoms recorded are described in online supplemental material 1.

STIssexually transmitted infections

### Prevalence of STI and co-infections

For FSWs providing all three samples (n=239), the prevalence of one or more STI tested at any anatomical site was 39.7% (95/239; 95% CI 33.8% to 46.1%) (table 2). Prevalence of one or more STI tested vaginally was 32.0% (80/250; 95% CI 26.5% to 38.0%), of which 58/80 (72.5%, 95% CI 61.9% to 81.1%) included TV. When limited to CT, NG and MG, which can infect all three anatomical sites, prevalence in vaginal, anorectal and oropharyngeal samples were 11.6% (29/250; 95% CI 8.2% to 16.2%), 19.7% (47/239; 95% CI 15.1% to 25.2%) and 3.2% (8/249; 95% CI 1.64% to 6.21%), respectively (p<0.001 for vaginal vs anorectal infections). Among those positive for STIs vaginally and anorectally, 10.0% (8/80; 95% CI 5.2% to 18.5%) and 19.2% (9/47, 95% CI 10.4% to 32.5%) had co-infections, respectively. There were no pathogen co-infections for oropharyngeal-positive samples (online supplemental table S2).

**Table 2:** Proportion of STIs overall and for each anatomical site in FSWs and NSWs

Infections	Overall*		Vaginal		Oropharyngeal		Anorectal
	FSWs (n=239)	NSWs(n=250)	FSWs(n=250)	NSWs(n=250)	FSWs(n=249)	NSWs(n=250)	FSWs(n=239)
	% (95% CI)	% (95% CI)	% (95% CI)	% (95% CI)	% (95% CI)	% (95% CI)	% (95% CI)
TV	23.4(18.5 to 29.2)	0.4(0 to 2.2)	23.2(18.4 to 28.8)	0.4(0 to 2.2)			
CT	12.6(8.9 to 17.4)	2.0(0.9 to 4.6)	4.0(2.2 to 7.2)	2.0(0.9 to 4.6)	1.6(0.6 to 4.1)	0.4(0 to 2.2)	11.3(7.9 to 15.9)
NG	6.7(4.2 to 10.6)	0.4 (0 to 2.2)	1.6(0.6 to 4.0)	0.4(0 to 2.2)	1.2(0.4 to 3.5)	0(0 to 0.2)	5.9(3.5 to 9.6)
MG	10.0(6.8 to 14.5)	3.2(1.6 to 6.2)	6.4(4.0 to 10.1)	3.2(1.6 to 6.2)	0.4(0 to 2.2)	0.4(0 to 2.2)	6.7(4.2 to 10.6)

* Overall status included only FSWs or NSWs providing all swabs from three or two anatomical sites, respectively

CT*Chlamydia trachomatis* FSWfemale sex workersMG*Mycoplasma genitalium*NG*Neisseria gonorrhoeae*NSWsnon-sex workersSTIsexually transmitted infectionsTV*Trichomonas vaginalis*

Among 250 NSWs providing swabs, the overall prevalence of having one or more vaginal and oropharyngeal STIs was 5.6% (14/250; 95% CI 3.4% to 9.2%) and 0.8% (2/251; 95% CI 0.2% to 2.9%), respectively (table 2). Only one vaginal sample had a pathogen co-infection, and no pathogen co-infections were found in oropharyngeal samples.

### Anatomical distribution of STIs

The anatomical distribution of CT, NG and MG infections among all FSWs are shown in online supplemental figure S1A–C. When both groups of FSWs were combined, for CT and NG most infections were detected only at the anorectum (62% (18/29; 95% CI 44.4% to 79.7%) and 68.8% (11/16; 95% CI 46.0% to 91.5%), respectively). For MG, vaginal and anorectal infections appeared to be more evenly represented.

Most STIs among NSWs were vaginal, with no infections detected only at the oropharynx.

### Vaginal cleaning and insertion practices

Overall, 87.2% (218/250; 95% CI 82.5% to 90.8%) of FSWs self-administered intravaginal cleaning and/or insertion products with one or more of alcohol, toothpaste, gentamycin cream, clotrimazole cream and/or antibiotic, antifungal steroid cream. For intravaginal cleaning, alcohol was most frequently used among FSWs (68.0% overall) compared with toothpaste among NSWs (online supplemental tables S3a and S3bSupplementary Materials 2, respectively).

### Condom use

Condom use for vaginal sex with clients was reported for 98.0% (245/250; 95% CI 95.4% to 99.1%). For 21.2% (53/250; 95% CI 15.9% to 25.8%) and 65.2% (163/250; 95% CI 59.1% to 70.8%) who reported having anal sex and oral sex with clients, 96.2% (51/53; 95% CI 87.3% to 99.0%) and 96.9% (158/163; 95% CI 93.0% to 98.7%) reported always using condoms, respectively. Of the 72.0% (180/250; 95% CI 66.1% to 77.2%) participants reporting ‘non-client partners’ that is, any other form of regular or casual partner 80.6% (145/180; 95% CI 74.2% to 85.7%) never used condoms for vaginal sex. For those having anal and oral intercourse with non-client partners, 78.8% (93/118; 95% CI 70.6% to 85.2%) and 80.6% (121/150; 95% CI 73.6% to 86.2%) reported never using condoms, respectively. Among NSWs 77.2% (173/224; 95% CI 71.3% to 82.2%) reported never using condoms for vaginal sex and 100% (222/222) of women who reported oral sex never used condoms (online supplemental tables S4a and S4b, respectively).

### Risk factor analysis

For vaginal infections, in univariate analysis, having an anorectal STI and not undertaking intravaginal insertion were significant factors. Age was also considered an important covariate for vaginal infection and maintained in the adjusted model. In multivariable analysis, having any anorectal STI (adjusted OR (aOR) (95% CI) 8.20, 3.78 to 18.6)) remained a significant predictor of vaginal infection (table 3). There was a suggestion that intravaginal insertion practices may have promoted the risk of vaginal STIs (aOR (95% CI) 0.5, 0.24 to 1.1). For anorectal infection, in univariate analysis STIs, having any vaginal CT, NG or MG (aOR (95% CI) 14.6, 5.6 to 41.8) or vaginal TV (aOR (95% CI) 2.9, 1.1 to 7.4) increased the risk of anorectal infection (table 4). Additionally, ‘having a non-client partner’ that is, any casual, regular or marital partner, decreased the risk of anorectal infection (aOR (95% CI) 0.4, 0.2 to 1.0).

**Table 3:** Risk factors associated with vaginal infection among brothel-based female sex workers

Characteristic	Univariate				Multivariable		
	N	OR	95% CI	P value	aOR	95% CI	P value
**Age**	205	1.00	0.96 to 1.04	0.98	1.01	0.97 to 1.05	0.62
Any anal STI	194						
Negative		Ref	—		—	—	
Positive		7.84	3.66 to 17.4	**<0.001**	8.20	3.78 to 18.6	**<0.001**
Any vaginal STI symptoms	205						
No symptoms		Ref	—				
Symptoms		1.61	0.78 to 3.54	0.21			
**No. of clients per week**	204	1.01	0.99 to 1.02	0.37			
Level of education	205						
Higher		Ref	—				
Primary		2.50	0.41 to 48.2	0.40			
Secondary		2.82	0.47 to 53.7	0.34			
Uneducated		1.40	0.05 to 41.6	0.83			
Condom use for vaginal sex (non-client partners)	205						
Always		Ref	—				
Not always		0.73	0.18 to 3.59	0.66			
No vaginal partner		0.71	0.17 to 3.68	0.66			
Condom use for anal sex (non-client partners)	205						
Always		Ref	—				
Not always		1.37	0.31 to 9.60	0.71			
No anal sex partner		1.24	0.28 to 8.66	0.80			
Having a non-client partner	205						
No		Ref	—				
Yes		1.04	0.54 to 2.10	0.90			
Use of self-medication	205						
Yes		Ref	—				
No		1.19	0.55 to 2.45	0.65			
Intravaginal cleaning	205						
Yes		Ref	—				
No		0.85	0.34 to 1.95	0.71			
Intravaginal insertion	205						
Yes		Ref	—		—	—	
No		0.58	0.31 to 1.09	0.092	0.50	0.24 to 1.01	0.056

Any P values presented in bold were signficant

aOR, adjusted ORSTIsexually transmitted infection

**Table 4:** Risk factors associated with anorectal STI among brothel-based female sex workers

Characteristic	Univariate				Multivariable		
	N	OR	95% CI	P value	aOR	95% CI	P value
**Age**	194	0.99	0.94 to 1.03	0.62			
Any vaginal CT, NG or MG	194						
Negative		Ref	—		—	—	
Positive		13.5	5.30 to 36.8	**<0.001**	14.6	5.55 to 41.8	**<0.001**
Vaginal TV infection	194						
Negative		Ref	—		—	—	
Positive		2.48	1.05 to 5.65	**0.033**	2.87	1.09 to 7.37	**0.029**
Any STI symptoms	194						
No symptoms		Ref	—				
Symptoms		1.96	0.81 to 5.51	0.16			
**No. of clients per week**	194	0.99	0.97 to 1.01	0.29			
Level of education	194						
Higher		Ref	—				
Primary		2.13	0.34 to 41.2	0.49			
Secondary		1.48	0.24 to 28.5	0.72			
Uneducated		2.33	0.07 to 74.5	0.59			
Condom use for vaginal sex (non-client partners)	194						
Always		Ref	—	—			
Not always		0.45	0.09 to 3.33	0.37			
No vaginal sex partner		0.98	0.19 to 7.29	0.98			
Condom use for anal sex (non-client partners)	194						
Always		Ref	—				
Not always		1.29	0.20 to 25.2	0.82			
No anal sex partner		1.65	0.26 to 32.0	0.65			
Having a non-client partner	194						
No		Ref	—		—	—	
Yes		0.49	0.24 to 1.03	0.058	0.43	0.18 to 1.00	**0.049**
Use of self-medication	194						
Yes		Ref	—				
No		1.03	0.41 to 2.38	0.94			
Intravaginal cleaning	194						
Yes		Ref	—				
No		0.54	0.15 to 1.49	0.28			
Intravaginal insertion	194						
Yes		Ref	—				
No		0.90	0.44 to 1.83	0.77			

Any P values presented in bold were significant

aOR, adjusted ORCT*Chlamydia trachomatis*MG*Mycoplasma genitalium*NG*Neisseria gonorrhoeae* STIsexually transmitted infectionTV*Trichomonas vaginalis*

## Discussion

In this study of FSWs in Ecuador, we demonstrated a high prevalence of vaginal TV, as well as CT and NG infections, detected only at the anorectum. Compared with anorectal infection, vaginal CT and NG proportions were much lower, but for MG we found an even distribution of infection vaginally and anorectally. Prevalence of oropharyngeal infections in FSWs was low but all infections were higher than those among our NSW population. Currently, there are few studies on anorectal STI prevalence among FSWs, but a report from Papua New Guinea demonstrated higher prevalence of both vaginal and anorectal STIs among FSWs.^21^ Although TV prevalence in our study was comparable to other FSW studies,^3^ other vaginal infections, particularly NG, were lower than previously described.^7^

Among those providing all swabs, the prevalence of at least one of the four STIs was nearly 40% and 12% of all FSWs had an infection detected only at the anorectum. When compared with sexually active clinical-attending women in high-income countries, studies have reported rates of >2% of isolated anorectal CT infection,^11^ suggesting our findings in Ecuadorian FSWs may be generalisable to other FSW communities globally. These studies also demonstrated high co-occurrence of vaginal and anorectal CT^11^ with little or no relationship between anorectal infection and reported anal sex.^22^ Similarly, in our study, virtually all FSWs reported consistent condom use for vaginal and anal sex with clients but inconsistent use with ‘non-client partners’. We were thus only able to examine the relationship of reported condom use with these non-client partners and STIs but found none. Our questionnaire, adapted from a previous one validated for condom use with ‘penetrative sex’, perhaps less accurately captured anal sex responses for various reasons, such as reluctance to disclose behaviours to healthcare workers, or fear of risky behaviours being discovered by brothel owners,^23^ perhaps explaining the high rates of infections detected only at the anorectum. Additionally, we found having ‘non-client partners’ indicated a decreased risk of anorectal STIs. It is possible individuals with ‘non-client partners’ may be more inclined to use condoms when working to avoid transmission to their partner. However, previous studies have documented an increased prevalence of STIs among FSWs that report having both client and non-client partners^24^ so further work is required to understand the relevance of our findings, particularly as we found no link with vaginal infection, or number of clients per week.

There are reasons why FSWs in brothels would consistently use condoms for vaginal sex, compared with anal sex, including preventing pregnancy^25^ and for avoiding genital infections, which if diagnosed may impact the ability to work within brothels. Studies have described substantial price premiums for those willing to provide condomless anal sex in Mexico and Ecuador,^26^ but work is needed to enhance the validity of reporting extragenital sex in FSWs. Among brothel-based FSW we did find a strong bidirectional association between vaginal and anorectal STIs, being in one anatomical location and having any STI in the other, suggesting that as well as condomless anal sex, contamination or autoinoculation between the vagina and anus may be important. However, we also found vaginal TV was a risk factor for having an anorectal STI, suggesting the high prevalence of anorectal infection may not only be due to contamination, but highlights sexual risk behaviours also being important. It is possible the high proportion of STIs detected only at the anorectum may have been influenced by lower-than-expected detection of vaginal co-infection, particularly given the low prevalence of vaginal CT and NG found. Vaginal cleansing practices, reported by over 80% of participants, is well documented among FSWs worldwide.^27^ It is plausible use of such cleansing products could interfere with PCR, but we found no evidence of PCR-inhibition and testing accuracy was quality-controlled by different testing methods (data not shown). Bactericidal properties of toothpaste, ethanol, gentamycin creams or vaginal suppositories could also plausibly impact bacterial load and thus detection of infection, but we found if anything, use of these appeared to promote vaginal infection. This could be an area of further research to assess the effectiveness of cleansing products and medicines for treating vaginal STIs against the risks of promoting infection or possible antimicrobial resistance, particularly for NG.

Among street-based FSWs in this study, TV prevalence was high and when compared with brothel-based workers, street-based FSWs were older (mean age 42 vs 26 years, respectively), earned less and were members of an FSW association. TV infection has been associated with older age, including in postmenopausal women, lower education level and high levels of poverty,^28^ aligning with our findings. We previously demonstrated an increased risk of STI acquisition for FSW association members and this appeared linked to FSW autonomy in contrast to ‘protected’ environments of brothels; FSW associations in these settings appear to primarily offer economic protection for FSWs, rather than health promotion and care.^16^

Our findings have the potential to impact STI control interventions and to provide STI transmission modelling parameters. Early STI detection and treatment, to reduce onward transmission, relies on regular testing across all relevant anatomical sites, for at-risk individuals. Many molecular diagnostics, test for just CT and NG, but also increasingly for TV. In our study, assuming the use of a highly accurate molecular test, 24 FSWs would need to be tested by VVS testing alone to detect one case of CT compared with 20 women if both VVS and oropharyngeal sites were sampled. This number decreases to eight FSWs with the addition of anorectal sampling. No anorectal sampling would miss 60% of FSWs infected with CT. Similarly, 60 FSWs would need to be tested to capture one NG infection if using VVS alone*,* 48 FSWs with the addition of oropharyngeal swabbing and 15 FSWs adding anorectal sampling. This makes a compelling case for evaluating, pooled multi anatomical-site testing approaches, which have previously demonstrated utility^29^ and which may result in significant reductions in STI detected per unit cost.^29 30^

### Strengths and limitations

Our study included results from understudied FSWs, from three different locations, in which data are limited for non-viral STIs and absent for extragenital infections. FSWs provided samples from three anatomical sites all collected in the same manner, ensuring robustness. All interviews were conducted in a private room to mitigate reporting inaccuracy.

There were several limitations to this work. First, NSWs only provided vaginal and oropharyngeal swabs, preventing comparisons of anorectal infection prevalence. Symptom data was limited to vaginal symptoms only and although data were captured on the use of antibiotics for sanitation practices, questionnaire answers for ‘Self-medication’ only captured the last ‘drug’ individuals used to manage any symptoms, frequently not antibiotics. We were unable to undertake a risk factor analysis for street-based FSWs as the small sample size was too small. Finally, we were unable to assess relative poverty as a risk factor, as the questionnaire did not include conventional indices of poverty, although all women were recruited from localities with very high levels of deprivation (see Methods).

## Conclusions

In conclusion, this study demonstrated a high prevalence of CT and NG infections detected only at the anorectum of FSWs, considered important populations for STI transmission and may guide improved estimates of STIs and inform on effective STI testing strategies. These data call for greater research into extragenital STIs in FSWs, including the role of vaginal self-medication and its potential impact on STI prevalence.

## supplementary material

10.1136/sextrans-2023-056075 online supplemental file 1

10.1136/sextrans-2023-056075 online supplemental file 2

10.1136/sextrans-2023-056075 online supplemental file 3

10.1136/sextrans-2023-056075 Abstract translation 1 This web only file has been produced by the BMJ Publishing Group from an electronic file supplied by the author(s) and has not been edited for content.

## Data Availability

All data relevant to the study are included in the article or uploaded as supplementary information.
