# Supplementary material for: Sexually transmitted infections among at-risk women in Ecuador: implications for global prevalence and testing practices for STIs detected only at the anorectum in female sex workers
Source: Sex Transm Infect. 2024 Aug 7;100(8):e056075. doi: 10.1136/sextrans-2023-056075 (PMC11672068; doi:10.1136/sextrans-2023-056075)
Supplement: online supplemental file 1 [file sextrans-100-8-s001.pdf]

SUPPLEMENTARY MATERIALS 1

Variable name	Description	Included for FSW or NSWs
FSW association	A local group or organisation that advocates rights and protection for workers, particularly those who undertake street-based work.	FSW
Vaginal symptoms	Can include one or more of the following: current discharge, lower abdominal pain, burning or itching, sores, lumps, or blisters in and around the vaginal and dyspareunia.	Both
Previous treatment for STI	Defined as any medication provided by a practitioner following symptoms suspected of an STI/diagnosis	Both
Self-medication	Use of one of antibiotics, analgesics, vaginal pessaries, or creams the last time they had vaginal symptoms	Both
Intravaginal cleaning	Washing around the vulva or inside the vaginal using a finger or cloth with products such as toothpaste or alcohol (purchased from a pharmacist), in the last six months	Both
Intravaginal insertion	Inserting or placing Gentamax (gentamycin cream), Trigenta (beclomethasone and nystatin), Canesten (clotrimazole cream or suppository) and leaving in the vagina regardless of duration, in the last six months	Both
Condom use	Use of a condom in vaginal, oral, and anal intercourse with clients and partners. Consistent condom use was defined as individuals always used condoms for that form of sexual practise, inconsistent condom use referred to any participants that may use condoms occasionally or frequently but not always	Both (questions regarding clients were only present in FSWs questionnaire)

Non-client partner	Any sexual contact, regular or casual in which the individual is engaging in sex not for the exchange of money or gifts.	FSW only
Pathogen co-infection	Presence of two or more of *C. trachomatis*, *N. gonorrhoeae*, *T. vaginalis*, or *M. genitalium* in one sample from one anatomical site. Anatomical co-infection was defined as one or more STIs in the vaginal, anorectal or pharynx of the same individual.	Both

Table A1: Description of variables

Swab taking

To collect a vulvo-vaginal samples a flocked swab was inserted approximately 2.5 cm inside the opening vagina and rotated for 15 sec., touching the walls of the vagina

PCR conditions

Reaction components were set up as follows: 5.0 µl of TaqMan™ Universal PCR Master Mix, 1.0µl 10x Exogenous Internal Positive Control (IPC) Mix, 0.2 µl of 50x IPC DNA, 1.05µl nuclease free water, 0.20µl primer mix (250nM per primer) and 0.05 µl probe (100nM) and 2.5 µl template or control DNA. All PCR reactions were run in repeats and had positive, negative and no-template controls for each run. All PCR reactions were run under the following cycling conditions: 95˚C for 10 min, followed by 40 cycles of 95°C for 15 s, 60°C for 1 min.

Luna cycling conditions

Reaction components were set up as follows: 1 µl primer mix (250nM per primer) and 0.4 µl probe (100nM) and 2.5 µl template DNA, made up to a total 20 µl with nuclease free water. All PCR reactions had positive, negative and no-template controls included. All PCR reactions were run under the following cycling conditions: 95˚C for 1 min, followed by 40 cycles of 95°C for 15s, 60°C for 30 seconds.
