# Supplementary material for: Sexually transmitted infections among at-risk women in Ecuador: implications for global prevalence and testing practices for STIs detected only at the anorectum in female sex workers
Source: Sex Transm Infect. 2024 Aug 7;100(8):e056075. doi: 10.1136/sextrans-2023-056075 (PMC11672068; doi:10.1136/sextrans-2023-056075)
Supplement: online supplemental file 2 [file sextrans-100-8-s002.pdf]

SUPPLEMENTARY MATERIALS 2

Table S1. Sociodemographic characteristics of NSWs recruited from the two recruited locations.

	Location A (n=157)	Location B (n=93)	Overall (n=250)	Differences observed between locations
**Sociodemographic characteristics**				
**Median age** (IQR)	31 (25-39)	29 (24-32)	30 (25-35)	P <0.001
**Country of origin (%)**				-
Ecuador	157 (100.0)	93 (100.0)	250 (100.0)	
Other	0 (0)	0 (0)	0 (0)	
**Level of education (%)****				P<0.001
Uneducated (Illiterate)	7 (4.5)	0 (0)	7 (2.8)	
Primary	77 (49.0)	28 (30.1)	105 (42.0)	
Secondary	67 (42.7)	55 (59.1)	122 (48.8)	
Higher	6 (3.8)	10 (10.8)	16 (6.4)	
**Number of Children (%)**				P = 0.013
NA	10 (6.4)	2 (2.2)	12 (4.8)	
0	0 (0)	2 (2.2)	2 (0.8)	
1-2	79 (50.3)	50 (53.7)	129 (51.6)	
3-5	54 (34.4)	37 (39.7)	91 (36.4)	
6-8	10 (6.4)	2 (2.2)	12 (4.8)	
9-10	4 (2.5)	0 (0)	4 (1.6)	
**Number of sexual partners in lifetime (%)**				P= 0.493
1-2	111 (70.7)	64 (68.8)	175 (70.0)	
3-5	39 (24.8)	27 (29.0)	66 (26.4)	
6-8	6 (3.8)	1 (1.1)	7 (2.8)	
9-10	0 (0)	0 (0)	0 (0)	
>10	1 (0.7)	1 (1.1)	2 (0.8)	
**Age first sexual intercourse (%)**				P = 0.438
1-5				
6-10	1 (0.7)	0 (0)	1 (0.4)	
11-13	2 (1.3)	0 (0)	2 (0.8)	
14-16	22 (14.0)	15 (18.3)	37 (14.8)	
17+	69 (43.9)	41 (44.1)	110 (44.0)	
	63 (40.1)	37 (37.6)	100 (40.0)	
**In employment (%)**				P = 0.522
Yes	43 (27.4)	29 (31.2)	72 (28.8)	
No	114 (72.6)	64 (68.8)	178 (71.2)	
**Clinical and behavioural characteristics**				
Any vaginal symptoms* (%)				P = 0.748
Yes				
No	134 (85.4)	77 (82.8)	211 (84.4)	
	23 (14.6)	16 (17.2)	39 (15.6)	
**Previous STI (in lifetime) (%)***				P = 0.446
Yes				
No	4 (2.5)	4 (4.3)	8 (3.2)	
	153 (97.5)	89 (95.7)	242 (96.8)	

**Intravaginal washing or douching** (%)				P = 0.424
Yes	51 (32.5)	36 (38.7)	87 (34.8)	
No	106 (67.5)	57 (61.3)	163 (65.2)	
**Intravaginal insertion (%)**				P = 0.004
Yes				
No	44 (28.0)	11 (11.8)	55 (22.0)	
	113 (72.0)	82 (88.2)	195 (78.0)	

*Further detailed information is described in Appendix 1 material
** Level of education refers to the percentage of individuals who attended school at each level, but may not have completed that stage

Table S2. Proportion of mono- and co-infections among FSW samples for each anatomical site

	Vaginal CT (n=10) N (%)	Vaginal NG (n=4) N (%)	Vaginal MG (n=16) N (%)	Vaginal TV (n=58) N (%)	Anorectal CT (n=27) N (%)	Anorectal NG (n=14) N (%)	Anorectal MG (n=16) N (%)
CT		0 (0)	1 (6.3)	3 (5.2)		0 (0)	5 (31.3)
NG	0 (0)		0 (0)	0 (0)	0 (0)		3 (18.8)
MG	1 (10)	0 (0)		4 (6.9)	5 (18.5)	3 (21.4)	
TV	3 (30)	0 (0)	4 (25.0)				
CT-NG			0 (0)	0 (0)			1 (6.3)
CT-MG		0 (0)		0 (0)		1 (7.1)	
NG-MG	0 (0)			0 (0)	1 (3.7)		
MG-TV	0 (0)	0 (0)					

Table S3a. Self-administered intravaginal cleaning and intravaginal insertion products in FSWs.

	**Location A (n = 63)**	**Location B (n = 142)**	**Location C (n = 45)**	**Overall (n = 250)**
**Alcohol (ethanol) (%)**	44 (69.8)	104 (73.2)	22 (48.8)	170 (68.0)
**Toothpaste (%)**	46 (73.0)	72 (50.7)	13 (28.8)	131 (52.4)
**Gentamycin cream (%)**	12 (19.0)	36 (25.4)	10 (22.2)	58 (23.2)
**Clotrimazole cream (%)**	17 (30.0)	43 (30.3)	16 (35.6)	76 (30.4)
**Neomycin, betamethasone and clotrimazole cream (%)**	7 (11.1)	20 (14.1)	6 (13.3)	33 (13.2)

Table S3b. Self-administered intravaginal cleaning and intravaginal insertion products in NSWs.

	Location A (n = 111)	Location B (n = 60)	Overall (n = 250)
**Alcohol (ethanol) (%)**	15 (9.6)	6 (6.5)	21 (8.4)
**Toothpaste (%)**	46 (29.3)	33 (35.5)	79 (31.6)
**Gentamycin cream (%)**	7 (4.5)	0 (0)	7 (2.8)
**Clotrimazole cream (%)**	34 (21.7)	7 (7.5)	41 (16.4)
**Neomycin, betamethasone and clotrimazole cream (%)**	18 (11.5)	4 (4.3)	22 (8.8)

Table S4a. Condom use for all types of sex with clients and non-client partners.

**Characteristic**	**Overall n=250**		**Location A (n = 63)**		**Location B (n = 142)**		**Location C (n = 45)**	
	**n**	**(%)**	**n**	**(%)**	**n**	**(%)**	**n**	**(%)**
**Condom use vaginal sex (all clients)**								
Always	245	98.0	62	98.4	140	98.6	43	95.6
Inconsistent	4	1.6	1	1.6	1	0.7	2	4.4
Never	1	0.4	----	----	1	0.7	----	----
**Condom use anal sex (all clients)**								
Always	51	20.4	21	33.3	27	19.0	3	6.7
Inconsistent	1	0.4	1	1.6	----	----	----	----
Never	1	0.4	----	----	----	----	1	2.2
No anal sex	197	78.8	41	65.1	115	81.0	41	91.1
**Condom use oral sex (all clients)**								
Always	158	63.2	47	74.6	93	65.5	18	40.0
Inconsistent	2	0.8	2	32.2	0	0	0	0
Never	3	1.2	0	0	2	1.4	1	2.2
No oral sex	87	34.8	14	22.2	47	33.1	26	57.8
**Condom use vagina sex partner (n=180)**								
Always	15	8.3	3	6.4	6	6.2	6	16.7
Inconsistent	20	11.1	8	17.0	11	11.3	1	2.8
Never	145	80.6	36	76.6	80	82.5	29	80.6
**Condom use anal sex partner (n=180)**								
Always	12	6.7	5	10.6	5	5.2	2	5.
Inconsistent	13	7.2	6	12.8	6	6.2	1	2.8
Never	93	51.7	23	48.9	53	54.6	17	47.2
No anal sex	62	34.4	13	27.7	33	33.0	16	44.4
**Condom use oral sex partner (n=180)**								
Always	15	8.3	4	8.5	7	7.2	4	11.1
Inconsistent	14	7.8	9	19.1	4	4.1	1	2.8
Never	121	67.2	27	57.4	72	74.2	22	61.1
No oral sex	30	16.7	7	14.9	14	14.4	9	25.0

Table S4b. Condom use for all types of sex with partners in NSWs.

Characteristic	Overall n=224		Location A (n = 140)		Location B (n = 84)	
	n	(%)	n	(%)	n	(%)
**Condom use vaginal sex partner**						
Always	6	2.7	2	1.4	4	4.8
Inconsistent	45	20.1	35	25.0	10	11.9
Never	173	77.2	103	73.6	70	83.3
**Condom use anal sex partner**						
Always	5	2.2	4	2.9	1	1.2
Inconsistent	11	4.9	9	6.4	2	2.4
Never	75	33.5	50	37.5	25	29.8
No anal sex	133	59.4	77	55.0	56	66.7
**Condom use oral sex partner**						
Never	222	100	140	100	82	100
