# Supplementary material for: Sexually transmitted infections among at-risk women in Ecuador: implications for global prevalence and testing practices for STIs detected only at the anorectum in female sex workers
Source: Sex Transm Infect. 2024 Aug 7;100(8):e056075. doi: 10.1136/sextrans-2023-056075 (PMC11672068; doi:10.1136/sextrans-2023-056075)
Supplement: online supplemental file 3 [file sextrans-100-8-s003.pdf]

Supplementary figure S1A-C: Anatomical distribution of CT, NG and MG infections among FSWs

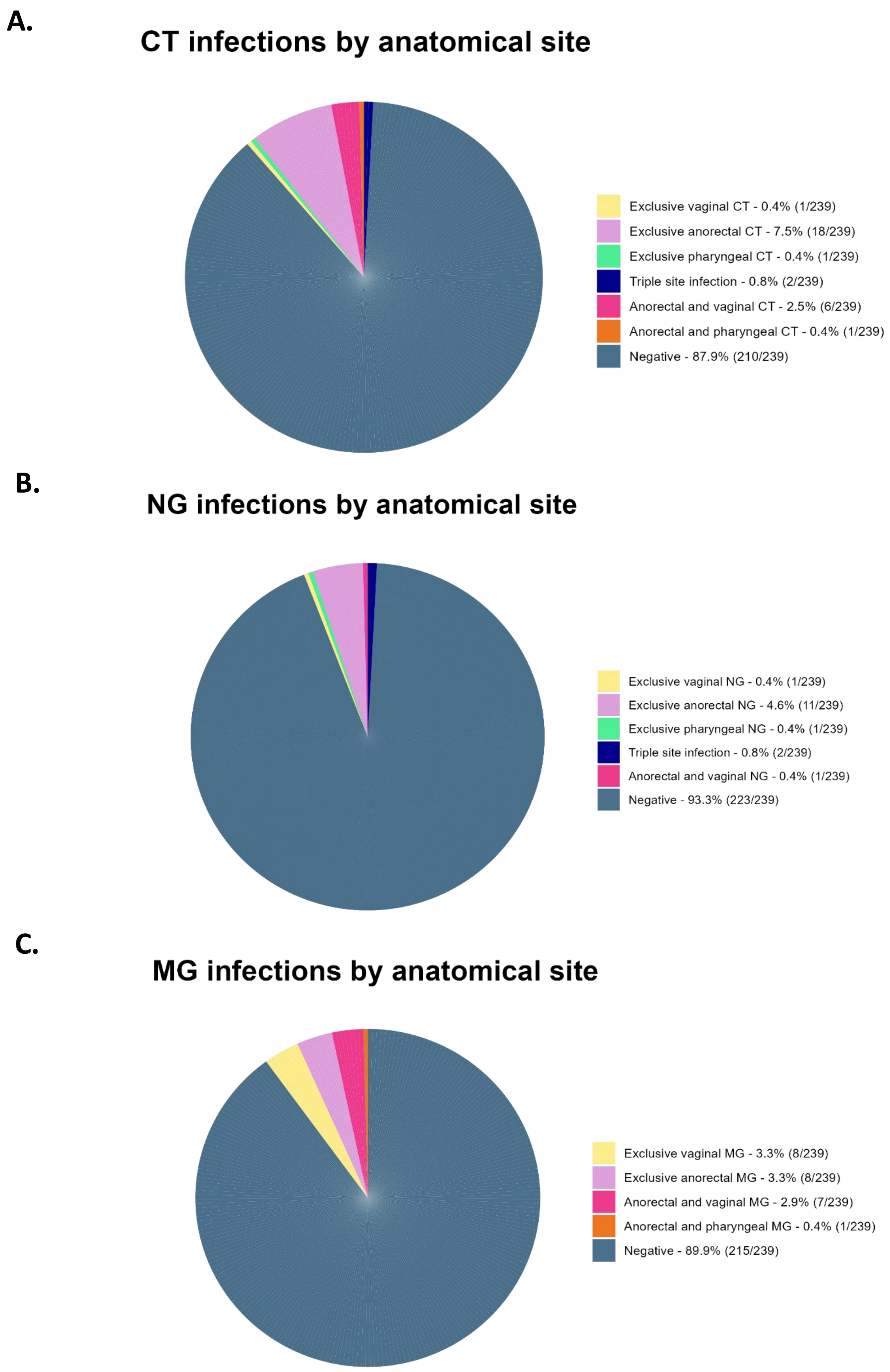
A.
CT infections by anatomical site
Exclusive vaginal CT - 0.4% (1/239)
Exclusive anorectal CT - 7.5% (18/239)
Exclusive pharyngeal CT - 0.4% (1/239)
Triple site infection - 0.8% (2/239)
Anorectal and vaginal CT - 2.5% (6/239)
Anorectal and pharyngeal CT - 0.4% (1/239)
Negative - 87.9% (210/239)
B.
NG infections by anatomical site
Exclusive vaginal NG - 0.4% (1/239)
Exclusive anorectal NG - 4.6% (11/239)
Exclusive pharyngeal NG - 0.4% (1/239)
Triple site infection - 0.8% (2/239)
Anorectal and vaginal NG - 0.4% (1/239)
Negative - 93.3% (223/239)
C.
MG infections by anatomical site
Exclusive vaginal MG - 3.3% (8/239)
Exclusive anorectal MG - 3.3% (8/239)
Anorectal and vaginal MG - 2.9% (7/239)
Anorectal and pharyngeal MG - 0.4% (1/239)
Negative - 89.9% (215/239)
