# Supplementary material for: Sexually transmitted infections among at-risk women in Ecuador: implications for global prevalence and testing practices for STIs detected only at the anorectum in female sex workers
Source: Sex Transm Infect. 2024 Aug 7;100(8):e056075. doi: 10.1136/sextrans-2023-056075 (PMC11672068; doi:10.1136/sextrans-2023-056075)
Supplement: Abstract translation 1 [file sextrans-100-8-s004.pdf]

Objetivos Las infecciones de transmisión sexual (ITS) anorrectales como *Chlamydia trachomatis* (CT) y *Neisseria gonorrhoeae* (NG) presentan desafíos en el tratamiento, pueden aumentar la resistencia a los antibióticos y, si no se detectan, pueden transmitirse a otras personas. Sin embargo, hay datos limitados a nivel mundial sobre la prevalencia de ITS anorrectales. Realizamos un estudio transversal para estimar la prevalencia y los factores de riesgo de ITS genitales y extragenitales no virales en mujeres trabajadoras sexuales (MTS) y mujeres no trabajadoras sexuales (MNTS) en Ecuador.

Métodos: 250 MTS adultas fueron reclutadas en sus lugares de trabajo (calle y burdeles) y 250 MNTS en tres localidades en el noroeste de Ecuador. Todas las participantes proporcionaron hisopos orofaríngeos y vulvovaginales (HVV), así como datos sociodemográficos. Las MTS también proporcionaron hisopos anorrectales. La detección de CT, NG, *Mycoplasma genitalium* (MG) en todos los hisopos, y *Trichomonas vaginalis* (TV) en los HVV se realizó mediante PCR. Los factores de riesgo se analizaron mediante regresión logística.

Resultados La prevalencia de infecciones vaginales, anorrectales y orofaríngeas en MTS fue del 32,0% (IC95%: 26,5%-38,0%), 19,7% (IC95%: 15,1%-25,2%) y 3,2% (IC95%: 1,6%-6,2%), respectivamente. La mayoría de las infecciones vaginales fueron por TV (23,4%; IC95%: 18,5%-29,2%). La prevalencia general de ITS en MTS, en cualquier sitio anatómico, fue del 39,7% (IC95%: 33,4%-45,4%), con un 12,1% (IC95%: 8,5%-16,9%) de infecciones anorrectales. De todas las infecciones por CT y/o NG, el 64,4% (IC95%: 50,4%-78,4%) fueron anorrectales. La prevalencia de ITS vaginal y orofaríngea en MNTS fue del 5,6% (IC95%: 3,4%-9,2%) y del 0,8% (IC95%: 0,2%-2,9%), respectivamente. La mayoría de las infecciones vaginales fueron por MG (3,2%; IC95%: 1,6%-6,2%). En el análisis multivariable, los factores de riesgo para contraer una ITS anorrectal en las mujeres trabajadoras sexuales que laboran en burdeles fueron las infecciones vaginales por CT, NG o MG ($p<0,001$), TV vaginal ($p=0,029$) y "tener pareja" ($p=0,038$).

Conclusiones: La alta prevalencia de infecciones anorrectales por CT y NG en mujeres trabajadoras sexuales sugiere que realizar únicamente pruebas genitales podría llevar a la omisión de infecciones significativas. Esto resalta la necesidad de investigar más a fondo el impacto en las estimaciones globales de ITS al no identificar infecciones extragenitales en mujeres en riesgo.
